# Response: Commentary: Why sprint interval training is inappropriate for a largely sedentary population

**DOI:** 10.3389/fpsyg.2016.00746

**Published:** 2016-05-19

**Authors:** Todd A. Astorino, Jacob S. Thum

**Affiliations:** Department of Kinesiology, California State University–San MarcosSan Marcos, CA, USA

**Keywords:** high intensity interval training, affect, enjoyment, exercise, perceptual responses

We have read Hardcastle et al. (2014) commentary questioning practicality of sprint interval training (SIT) in untrained populations. We have identified key areas which were previously overlooked (Del Vecchio et al., 2015; Jung et al., 2016) which may augment this discussion concerning feasibility of SIT.

## Sprint interval training is not “*one size fits all*”

One aspect previously ignored is the diversity of approaches used to administer SIT. Initially, SIT was instituted using repeated Wingate-tests which are quite impractical in sedentary adults. However, this regime was well-tolerated in obese men (Whyte et al., 2010) and sedentary, overweight women (Trilk et al., 2011). In untrained adults, SIT was completed at intensities from 120 (Matsuo et al., 2014) to 170%Wmax (Gillen et al., 2014), with the latter study requiring completion of 3 min/week of SIT for 6 week. These protocols are less strenuous than “Wingate-style” SIT performed at work rates of 300%Wmax (Burgomaster et al., 2008; Astorino et al., 2011). Across studies, maximal oxygen uptake (VO_2_max) was significantly increased, demonstrating the health benefits of SIT despite lower workloads performed during training.

Regimes of SIT differ in the magnitude of acute changes in physiological and perceptual function. Wood et al. (2016) compared metabolic and perceptual responses between SIT (eight bouts at 130%Wmax separated by 90 s recovery) and HIIT (eight bouts at 85%Wmax separated by 75 s recovery) in active adults. Oxygen uptake (VO_2_) increased up to 90%VO_2_max during HIIT, which was higher than in SIT. Rating of perceived exertion and blood lactate concentration (BLa) were lower in HIIT. Nevertheless, affect was similar between regimes and 50% of participants preferred HIIT and 50% preferred SIT. Nevertheless, perceptions of SIT were not compared to continuous exercise (CEX), so it is unknown if these sensations are more aversive than HIIT or heavy CEX, which was the least preferred mode of exercise in untrained adults (Jung et al., 2014).

A few studies have compared chronic adaptations between HIIT and SIT, with some data (Zelt et al., 2014; Foster et al., 2015) showing similar adaptations; whereas, others (Matsuo et al., 2014; Bækkerud et al., 2016) demonstrated that higher-volume HIIT elicits greater increases in VO_2_max than SIT or CEX. Sloth et al. (2013) showed 4–13% increases in VO_2_max after chronic SIT. Discrepancies between studies are attributed to methodological differences as well as individual variability in VO_2_max response to training (Astorino and Schubert, 2014).

## Is sprint interval training enjoyable?

We acknowledge that any mode of training including SIT has little application to adults if it is inaccessible or poorly-tolerated. This is especially true because most adults are not habitually active. Preliminary data in active men and women with spinal cord injury (Thum and Astorino, 2015) demonstrate higher exercise enjoyment during HIIT or SIT vs. CEX. In this study, exercise enjoyment measured with the Physical Activity Enjoyment Scale (PACES; Kenzierski and Dicarlo, 1991) was determined 10 min after completion of CEX (25 min at 45%Wpeak), HIIT (eight 60 s bouts at 70%Wpeak), and SIT (eight 30 s bouts at 105%Wpeak). Despite significantly higher BLa and less positive affect in HIIT and SIT, enjoyment was significantly higher by 22–26 units in HIIT and SIT vs. CEX. These data support findings in inactive populations (Jung et al., 2014, Martinez et al., 2015) and oppose the Dual-Mode theory (Ekkekakis et al., 2011) which posits that exercise above the ventilatory threshold characteristic of SIT and HIIT elicits more unpleasant feelings than CEX. Nevertheless, interval training is not continuous but provides the exerciser with recovery between bouts which may improve perceptions of exercise and subsequently exercise enjoyment. Tritter et al. (2013) reported that self-efficacy declined during an acute bout of SIT, yet this decline was minimized by positive feedback given before exercise. Moreover, enjoyment was higher in response to positive feedback vs. negative feedback. Tempest and Parfitt (2016) showed that greater tolerance to intense exercise as measured with a questionnaire led to more positive affective responses and different hemodynamic responses in the prefrontal cortex compared to individuals with lower tolerance. Whether greater tolerance to SIT leads to less aversive affective responses and greater adherence is unknown.

## Are people likely to adhere to sprint interval training?

Despite the robust physiological adaptations observed with SIT, it is unknown if individuals will adhere to it long-term. Lunt et al. (2014) randomized sedentary, overweight to obese adults to 12 week of walking, aerobic interval training (AIT), or maximal volitional interval training (MVIT) consisting of walking or jogging at various fractions of maximal heart rate (HRmax) which were performed in an outdoor group setting. Data showed modest changes in VO_2_max, which was explained by poor adherence to training. Although, attendance was similar (75%) in participants performing walking or MVIT, and adherence to AIT was lower (59%). These findings question the feasibility of group-based HIIT conducted outside of a lab for significantly improving cardiorespiratory fitness. Over a 1 month period, pre-diabetics showed greater adherence to HIIT vs. CEX when it was performed in a free-living state (Jung et al., 2015). This is an important topic considering that greater adherence to vigorous physical activity may optimize resultant changes in health and fitness.

## Conclusion

We credit Hardcastle et al. (2014) for their initiative in inspiring a lively exchange of ideas. However, the assertion that SIT/HIIT is not a viable exercise modality for sedentary individuals is not supported by empirical data. These protocols are extremely malleable and therefore compatible to individuals of diverse fitness and motivation. However, considering the psychological responses of long-term SIT performed in and outside of a lab setting would be an important element in predicting adherence to this exercise modality. Such evaluation may lead to a greater understanding of the realistic health benefits of SIT before it is dismissed as an elite form of exercise relegated solely to active men and women.

## Author contributions

TA developed the idea for this commentary, wrote the majority of the text, and approved the final version. JT also wrote part of the commentary, assisted in the revision of the commentary and responses to the reviewers, and approved the final version.

### Conflict of interest statement

The authors declare that the research was conducted in the absence of any commercial or financial relationships that could be construed as a potential conflict of interest.
